# Expression and Clinical Significance of Activating Transcription Factor 3 in Human Breast Cancer

**Published:** 2013-11

**Authors:** Hua Cao, Zhi-Xue Yang, Guo-Qin Jiang

**Affiliations:** 1 Department of General Surgery, the Second Affiliated Hospital of Soochow University, Suzhou, Jiangsu, China

**Keywords:** Activating transcription factor 3 (ATF3), Breast cancer, Clinical significance, Immunohistochemistry, Prognostic indicator

## Abstract

***Objective(s):*** Breast cancer is the most common type of cancer among women worldwide. This study investigated the expression and clinical significance of activating transcription factor 3 (ATF3) in human breast cancer and its relationship with the clinical outcome of breast cancer.

***Materials and Methods*** **:** ATF3 expressions were detected in 114 primary breast cancer tissues and 114 adjacent normal tissues using immunohistochemistry (IHC) assay. Categorical variables were statistically compared by chi-square or Fisher’s exact test. Survival curves were evaluated using the Kaplan-Meier method and comparisons of survival rates were tested using a Log-rank test.

***Results*** **:** IHC analysis showed that the positive expression of ATF3 protein was detected in breast cancer tissue with a positive ratio of 76.3%, and the positive ATF3 expression in adjacent normal breast tissue was 13.2%, which is lower than that in breast cancer tissue samples (*P*<0.01). Furthermore, ATF3 expression showed significant correlation with TNM stage, invasion, lymph node metastasis and number of metastatic lymph nodes (*P*=0.038, *P*=0.029, *P*=0.026, and *P*=0.039 respectively), and did not correlate with patients’ age and tumor size (*P*>0.05). A significant difference in overall survival rate was found between the patients with positive expression of ATF3 protein and those with negative expression (*P*=0.041).

***Conclusion*** **:** Increased ATF3 expression participate in the tumorigenesis, invasion and metastasis of breast cancer, and ATF3 may be useful as a new prognostic indicator for breast cancer patients.

## Introduction

Breast cancer is the most common type of cancer among women worldwide, and the incidence of breast cancer is increasing in the developing world (1). Risk factors for breast cancer include repro-ductive factors associated with prolonged exposure to endogenous estrogens, genetic factors, lifestyle factors, and others (1, 2). Activating transcription factor 3 (ATF3) is a member of the ATF/CREB family of transcription factors, which shares the basic region leucine zipper DNA binding motif and binds to the ATF/CRE consensus sequence TGACGTCA(3-5). Overwhelming evidence indicates that *ATF3* gene expression may be upregulated by a variety of stress signals during their development (6, 7). However, until recently, there have been no studies investigating the clinical significance of ATF3 in human breast cancer. 

In this study, we collected 114 cases of breast cancer and 114 cases of adjacent non-tumor breast tissues, and explored the expression of ATF3 protein to determine whether or not ATF3 expression influences breast cancer malignancy and clinical outcome.

## Materials and Methods

***Patients***

A total of 114 patients who underwent potentially curative resection for breast cancer at the Department of General Surgery, the 2^nd^ Affiliated Hospital of Soochow University from January 2009 to December 2011 were eligible for this study, ranging in age from 24 to 88 years (median age 53.5 years) (Table 1). There are 15 intraductal carcinoma, 42 infiltrative ductal carcinoma, 44 infiltrative lobular carcinoma, and 13 other infiltrative carcinoma.

Eligibility criteria for this study included: 1) histologically proven primary breast cancer, 2) no history of mastectomy or other malignancy, 3) a lack of noncurative surgical factors except for distant metastasis (such as liver, lung, brain, or bone-marrow metastasis) and supraclavicular lymph node metastasis, 4) axillary lymph node dissection (ALND) performed, and 5) no patients died during the initial hospital stay or for 1 month after surgery, 6) no neoadjuvant therapy (including chemotherapy, radiotherapy, and hormone therapy). 

**Table 1:** Relationship between the expression of ATF3 and the clinical pathological characteristics in breast cancer tissues

Characteristic	n	Expression of ATF3		*P* *-* value
		Positive (%)	Negative (%)	
Age (yr)				0.871
≤50	48	36 (75.0)	12 (25.0)	
>50	66	51 (77.3)	15 (22.7)	
Primary tumor size (cm)				0.206
≤2	18	9 (50.0)	9 (50.0)	
>2, ≤5	90	72 (80.0)	18 (20.0)	
>5	6	6 (100.0)	0 (0.0)	
TNM stage*				0.038
I-II	46	28 (60.9)	18 (39.1)	
III-IV	68	59 (86.8)	9 (13.2)	
Invasion				0.029
No	15	3 (20.0)	12 (80.0)	
Yes	99	82 (82.8)	17 (17.2)	
Lymph node metastasis				0.026
No	28	11 (39.3)	17 (60.7)	
Yes	86	75 (87.2)	11 (12.8)	
Number of metastatic lymph nodes				0.039
0	28	11 (39.3)	17 (60.7)	
1-3	29	15 (51.7)	14 (48.3)	
4-9	37	30 (81.1)	10 (18.9)	
≥10	20	19 (95)	1 (5)	

ATF3: activating transcription factor 3; *According to the 7^th^ AJCC (American Joint Committee on Cancer) TNM classification for breast cancer

***Immunohistochemistry***

Tissue specimens of primary breast cancer samples and adjacent normal tissue samples from 114 patients were fixed in 10% formalin and paraffin-embedded according to standard procedures. Tissue sections of 4 μm were prepared, and endogenous peroxidase activity was blocked by incubation in 0.5% H_2_O_2_ for 10 min. Microwave antigen retrieval was performed in 0.1 mol/l citrate buffer (pH 6.0) at 750 W for 10 min. To reduce nonspecific binding, the slides were incubated with 1% normal goat serum for 30 min at room temperature. After washing three times with phosphate buffered saline (PBS), the slices were incubated with mouse anti-human ATF3 antibody (1:50; Abcam) at 4°C overnight. After centrifuging three times with PBS, the specimens were incubated with mouse secondary antibody (1:200; DAKO, Canada) at room temperature for 1 hr. Finally, the centrifuged slices were stained with diaminobenzidine (DAB) as a chromogen. PBS-only stained sample was used as a background control.

The grade of staining was determined independ-ently by two experienced pathologists. Staining intensity (0 to 3: weakly intense to strongly intense) and the proportion of stained cells (0 to 3: no cells stained to more than 50% cells stained) were determined as described. A cumulative score of ≥3 was considered to be positive expression.

***Follow-up***

After curative surgery, all patients were followed every half a year or until death. The median follow-up time was 31 months (range, 13-48 months). The follow-up of all patients who were included in this study was completed in January 2013.

***Statistical analysis***

All statistical analyses were performed with statistical analysis program package SPSS version 18.0 (SPSS Inc., Chicago, USA). Categorical variables were statistically compared with chi-square or Fisher’s exact test. Survival curves were evaluated using Kaplan-Meier method and comparisons of survival rates were tested using a Log-rank test. A P value of less than 0.05 was considered statistically significant.

**Figure 1:**
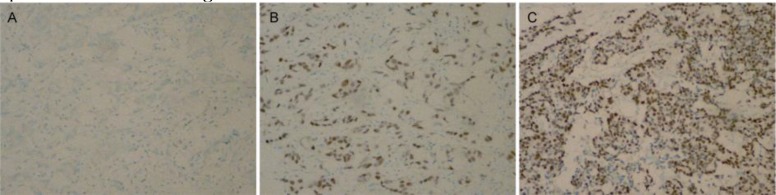
Detection of ATF3 expression in breast cancer tissues and adjacent normal tissues by immunohistochemistry assay (DAB, ×40). A: Negative control; B: Adjacent normal tissues sample; C: Breast cancer tissues sample

## Results

***E*** ***xpression of ATF3 in breast cancer*** ***and adjacent normal breast*** ***tissues***

ATF3 expression was undetectable in the negative control samples (Figure 1A) and ATF3 protein was weakly expressed mostly in the adjacent normal breast tissues (Figure 1B), however, in breast cancer cells, there was a remarkable increase (Figure 1C). ATF3 was observed in the nuclei of breast cancer cells and typically appeared as buffy granulo-staining (Figure 1). The positive expression rate of ATF3 was 76.3% (87/114) in breast cancer tissues, whereas the rate was 13.2% (15/114) in adjacent normal breast tissues. The difference in ATF3 expression between breast cancer and adjacent normal breast tissues was statistically significant (*P*=0.009).

***Relation*** ***ship*** ***between the expression of ATF3 and the clinical pathological characteristics in breast cancer*** ***tissues***

Positive expression rate of ATF3 was significantly correlated with TNM stage (*P*=0.038), invasion (*P*=0.029), lymph node metastasis (*P*=0.026), and number of metastatic lymph nodes (*P*=0.039), but not with patients’ age and primary tumor size (*P*>0.05, Table 1). As TNM stage and number of metastatic lymph nodes increased, the positive expression rate of ATF3 was higher.

***Relation*** ***ship*** ***between*** ***ATF3 expression and survival***

**Figure 2:**
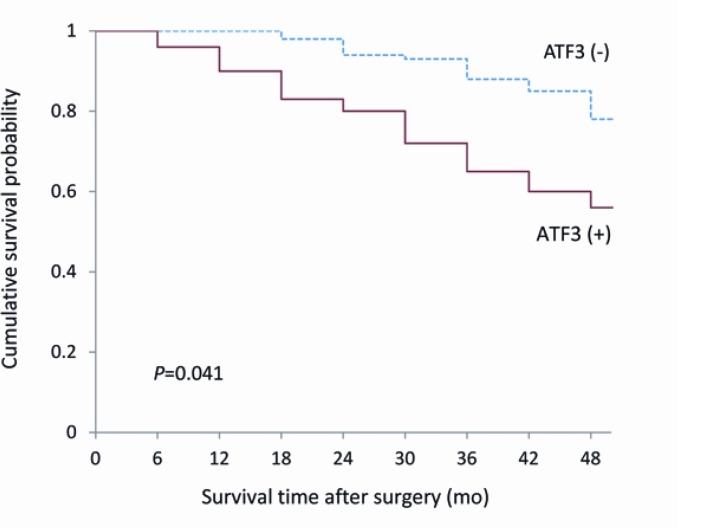
Kaplan-Meier survival curve for ATF3 expression (+) and ATF3 expression (–) breast cancer patients. *P*=0.041, Log-rank test

Among 114 breast cancer patients, 70 patients are alive, 31 patients died of distant metastases and 13 others died of local regional recurrence. As for the Kaplan-Meier survival analysis (Figure 2), a signi-ficant difference in overall survival rate was found between the patients with positive expression of ATF3 protein and those with negative expression (*P*=0.041). 

## Discussion

ATF3 belongs to the ATF/cyclic AMP response element binding (CREB) family of transcription factors, and most normal cells have very weak or absent ATF3 expression under steady-state conditions (8-10). *ATF3* is activated in most human cancers either directly or indirectly, which highlights its critical biological function as a tumor suppressor gene. However, significant upregulated ATF3 expression can be observed when cell stress is induced, which implies that *ATF3* is a universal “adaptive response gene” (11, 12). In normal tissues, ATF3 may promote cell proliferation and cell apoptosis. In contrast, it has been identified in neoplasms as either an oncogene or as tumor suppressor, depending on tumor entity and grade. For example, ATF3 can mediate pro-apoptotic effects in human mammary epithelial cells. However, it may promote cell survival, motility, and invasiveness in breast cancer cells. Transgenic mice that over-express ATF3 in basal epithelial cells develop dysplastic lesions, epidermal hyperplasia, and oral squamous cell carcinoma (4, 13). Also in favor of oncogenicity, the tumor suppressor gene *Drg-1* mediates its anti-metastatic properties through ATF3 downregulation in prostate cancer (4, 14, 15). Up to now, to the best of our knowledge, the relationship and molecular mechanisms of ATF3 in the progression and metastasis of breast cancer remain unclear. It is now necessary to elucidate the relationship between ATF3 and breast cancer based on the results of previous studies. We found that the ATF3 expression was markedly increased in breast cancer tissues compared to adjacent normal breast tissues (*P*<0.01), and ATF3 expression correlated with TNM stage, invasion, lymph node metastasis and number of metastatic lymph nodes (*P*=0.038, *P*=0.029, *P*=0.026, and *P*=0.039, respectively). Moreover, as TNM stage and number of metastatic lymph nodes increased, the positive expression rate of ATF3 was higher. This indicated that ATF3 might be involved in the tumorigenesis, invasion and metastasis of breast cancer, inferring that ATF3 might be a new tumor marker for breast cancer patients. A significant difference in overall survival rate was also found between the patients with positive expression of ATF3 protein and those with negative expression (*P*=0.041), suggesting that ATF3 may be a new prognostic indicator for breast cancer patients. In view of these data, ATF3 may play an oncogenic role in human breast tumorigenesis and development. However, our study has given limited data about role of ATF3 in breast cancer, and the diagnostic, predictive and prognostic value of ATF3 and its mechanism needs to be investigated thoroughly. Further studies on a large scale should be performed, and they might support the idea that ATF3 is useful as either a biomarker or therapeutic target of breast cancer, since breast cancer has been recognized as a heterogeneous disease (16).

## Conclusion

Our data indicated ATF3 might be involved in the tumorigenesis, invasion and metastasis of breast cancer, and increased ATF3 expression may lead to poor prognosis, which suggests that ATF3 may play an oncogenic role in human breast tumorigenesis and development, and is useful as either a biomarker or therapeutic target of breast cancer.
